# Best Evidence Rehabilitation for Chronic Pain Part 3: Low Back Pain

**DOI:** 10.3390/jcm8071063

**Published:** 2019-07-19

**Authors:** Anneleen Malfliet, Kelly Ickmans, Eva Huysmans, Iris Coppieters, Ward Willaert, Wouter Van Bogaert, Emma Rheel, Thomas Bilterys, Paul Van Wilgen, Jo Nijs

**Affiliations:** 1 Research Foundation–Flanders (FWO), 1090 Brussels, Belgium; 2 Department of Physiotherapy, Human Physiology and Anatomy (KIMA), Faculty of Physical Education & Physiotherapy, Vrije Universiteit Brussel, 1090 Brussels, Belgium; 3 Pain in Motion International Research Group, 1090 Brussels, Belgium; 4 Department of Physical Medicine and Physiotherapy, University Hospital Brussels, Laarbeeklaan 101, 1090 Brussels, Belgium; 5 Department of Rehabilitation Sciences and Physiotherapy, Faculty of Medicine and Health Sciences, Ghent University, 9000 Gent, Belgium; 6 Department of Public Health (GEWE), Faculty of Medicine and Pharmacy, Vrije Universiteit Brussel, 1090 Brussels, Belgium; 7 Department of Experimental-Clinical and Health Psychology, Ghent University, 9000 Ghent, Belgium; 8 Transcare, Transdisciplinary Pain Management Centre, 9728 EE Groningen, The Netherlands

**Keywords:** pain neuroscience, musculoskeletal pain, rehabilitation medicine, physiotherapy, lifestyle

## Abstract

Chronic Low Back Pain (CLBP) is a major and highly prevalent health problem. Given the high number of papers available, clinicians might be overwhelmed by the evidence on CLBP management. Taking into account the scale and costs of CLBP, it is imperative that healthcare professionals have access to up-to-date, evidence-based information to assist them in treatment decision-making. Therefore, this paper provides a state-of-the-art overview of the best evidence non-invasive rehabilitation for CLBP. Taking together up-to-date evidence from systematic reviews, meta-analysis and available treatment guidelines, most physically inactive therapies should not be considered for CLBP management, except for pain neuroscience education and spinal manipulative therapy if combined with exercise therapy, with or without psychological therapy. Regarding active therapy, back schools, sensory discrimination training, proprioceptive exercises, and sling exercises should not be considered due to low-quality and/or conflicting evidence. Exercise interventions on the other hand are recommended, but while all exercise modalities appear effective compared to minimal/passive/conservative/no intervention, there is no evidence that some specific types of exercises are superior to others. Therefore, we recommend choosing exercises in line with the patient’s preferences and abilities. When exercise interventions are combined with a psychological component, effects are better and maintain longer over time.

## 1. Introduction

Chronic Low Back Pain (CLBP) is a major health problem worldwide and prevalence numbers have increased substantially in the past decades [1]. A global systematic review reports a linear correlation between age and CLBP prevalence, more specifically, individuals aged between 20 and 59 have a CLBP prevalence of 19.6%, while the prevalence in older people is 25.4% [2]. Besides pain, disability is reported very frequently. CLBP is a major contributor to the global disability burden, and continues to be the leading cause of years lived with disability [3,4]. About half of the people who experience LBP will seek care [5]. Given the high prevalence numbers of CLBP [2], this relates to excessive direct and indirect health care costs as well as a major social and economic impact [6,7]. 

Current guidelines recommend non-pharmacological and non-invasive management, including the advice to stay active, the use of patient education and exercise therapy [8]. Yet, given the high number of treatment guidelines, systematic reviews and randomized controlled trials on CLBP management, clinicians might be overwhelmed by the evidence available. Taking into account the scale and costs of the CLBP problem, it is imperative that healthcare professionals involved in CLBP management should have access to up-to-date, evidence-based information to assist them in treatment decision-making. Therefore, this paper aims to endorse consistent best practice, to reduce unwarranted variation and to diminish the use of low-value interventions in CLBP care.

Here, a state-of-the-art overview of the best evidence non-invasive rehabilitation for people having CLBP is provided. The best evidence non-invasive rehabilitation is reviewed in a way that clinicians can integrate the evidence into their daily clinical routine. In addition, the state-of-the-art overview also serves clinical researchers to build upon the best evidence for designing future trials and implementation studies, and to develop new innovative studies. 

## 2. State of the Art

To cover the best evidence non-invasive rehabilitation, this section relies on systematic reviews and meta-analyses primarily. A non-systematic search of the literature was performed in PubMed, Web of Science and Google Scholar using the following search terms: rehabilitation, chronic low back pain, chronic back pain, chronic lumbar pain, chronic lower back pain. When possible, we used ‘systematic review’, and ‘meta-analysis’ filters. Additionally, information from several international clinical guidelines was retrieved and discussed.

Given the strong empirical support indicating that pain severity alone is not a robust predictor of function and improvement, we will focus both on pain and function as outcomes for chronic low back pain management [9,10]. 

### 2.1. Evidence from Systematic Reviews, and Meta-Analyses 

A non-systematic search for evidence on non-invasive rehabilitation modalities for CLBP increases the understanding that CLBP is not only a common health problem but is also highly investigated. Unfortunately, many systematic reviews focus on LBP in general, and include both (sub)acute and chronic LBP. When the results of both populations were merged together in a review and specific conclusions for CLBP could not be identified, these papers were excluded from this overview. An outline of the available systematic reviews and meta-analyses that focused solely on CLBP, or in which CLBP results could be isolated, can be found in Table 1. If more than one systematic review was found regarding a specific topic, priority was given to including a meta-analysis (if available) and/or the most recent paper available. 

The overview of evidence available from systematic review and meta-analyses is presented using the subdivision based on physically ‘*active*’ and ‘*inactive*’ interventions. Yet, this subdivision is chosen for practical reasons, and relies on whether an intervention requires the patient to be physically active or not. Therefore, pain neuroscience education will be discussed as part of the physically inactive interventions. Yet, we would like to stress that pain neuroscience education requires mental and cognitive activity of the patient given the required interaction between patient and therapist.

### 2.2. Physically Inactive Interventions

Investigated inactive techniques for CLBP include therapeutic ultrasound, kinesiotape, pain neuroscience education, transcutaneous electrical nerve stimulation, massage, osteopathic intervention, and spinal manipulative therapy (including high-velocity low-amplitude spinal manipulations as well as low-velocity low-amplitude mobilizations). Out of these therapies, only two are recommended, and only when implemented as adjunctive therapy: pain neuroscience education and spinal manipulative therapy. All other inactive interventions (i.e., therapeutic ultrasound, kinesiotape, transcutaneous electrical nerve stimulation, massage and osteopathic interventions) are not recommended for CLBP management based on available evidence. 

Pain neuroscience education aims to decrease the threat value of pain by increasing the patient’s knowledge about pain and by reconceptualizing pain [38]. As stand-alone intervention, this treatment modality can reduce disability and kinesiophobia short term, but is not able to change pain [13]. However, when combined with other physiotherapeutic interventions, pain neuroscience education can significantly reduce pain short term [13]. Therefore, pain neuroscience education can be considered as a first step before applying an active intervention for people with CLBP. Given that many people with CLBP display kinesiophobia (i.e., fear of movement and avoidance behavior, which is a barrier for positive treatment outcome) [39], and active interventions are recommended (see below: ‘active interventions’ and ‘international guidelines’) [40], pain neuroscience education can prime people for further treatment by adapting beliefs and expectations. We would like to emphasize that—although here discussed among physically inactive interventions—pain neuroscience education requires a certain degree of activity of the patient. Pain neuroscience education should be delivered using intense interaction between the patient and the therapist, and therefore requires mental and cognitive activity of the patient [41,42]. Additionally, pain neuroscience education appears to enhance physical activation and its effects on pain, given the evidence that combining pain neuroscience education with a (therapeutic) exercise intervention is more effective than an exercise intervention alone (large effect size for pain intensity) [13,43]. Manuals to implement pain neuroscience education are available in books [44,45], and tools for clinical practice can be found online [46].

Similarly, spinal manipulative therapy can be used in clinical practice for CLBP management, but only as part of a treatment package (i.e., adjunctive therapy) given the moderate quality evidence from improvements in pain and function at short-term follow-up (1 month) but not at long term (6 or 12 months follow-up) [18,40,47,48]. Importantly, evidence reports several possible adverse events related to spinal manipulative therapy, which should be taken into account by the clinician before using these techniques. Reported adverse events include severe back pain, acute flare-up of back pain, inability to sleep because of pain, muscle soreness and stiffness, exacerbation of symptoms, and tiredness [18]. Interestingly, a randomized controlled trial examined if the effects on pain differed between region-specific and non-region-specific spinal manipulations in people with CLBP (*n* = 148) [49]. While both groups showed a reduction in pain intensity after the manipulation, they did not find any differences between region-specific and non-region-specific techniques. This finding appears to refute any local, biomechanical mechanisms behind the effectiveness of these techniques [49]. Changes in pain in response to manipulative techniques in people with CLBP could therefore be more related to a cascade of neurophysiological responses from both the peripheral and central nervous system as well as nonspecific effects such as expectations and psychosocial factors, rather than local tissue changes [49]. 

As the effects of spinal manipulative techniques in CLBP might be explained by similar mechanisms contributing to the positive effects of pain neuroscience education, some researchers suggest to combine both recommended physically inactive adjunctive therapies discussed here [50,51]. Given their similar recommendation (i.e., as adjuvant therapy to active treatment modalities), discussing their simultaneous application in clinical practice becomes relevant. Because of the aim of pain neuroscience education to shift the patient’s focus away from the tissues in the low back as the source of their pain, many could conclude that pain neuroscience education should be used solely within a hands-off treatment approach. Yet, the meta-analysis of Wood et al. (2018) includes papers that combine pain neuroscience education with other physiotherapeutic interventions such as exercise/activity and/or manual therapy [13]. Outcomes appeared to favor the combination of pain neuroscience education with movement, either passive (manual therapy) and/or active. This suggests that combining pain neuroscience education with “hands-on” approaches results in more favorable responses than pain neuroscience education alone [51]. Yet, given the statement of The American Physical Therapy Association (APTA) that warns us for the negative effects of applying physically inactive treatments (i.e., they can delay recovery and lead to poor long-term outcomes by reinforcing a passive role, promoting inactivity and disability behavior, and ‘medicalizing’ the patient), combining pain neuroscience education with active exercise therapy might still be preferred over any physically inactive approach. 

If a clinician were to combine pain neuroscience education with “hands-on” techniques, care should be taken that all communication to the patient fits within the biopsychosocial framework of PNE. Therefore, it should be avoided to present manual techniques within a biomedical pain model, in which the therapist is deemed to “fix” a structure [52,53]. Instead, communication can focus on the desensitizing effects of “hands-on” techniques and threatening words such as “pain” can be replaced by more neutral terms like “symptoms” [52,54]. 

### 2.3. Physically Active Interventions

Given the listed active interventions in Table 1 and their recommendations, physically active interventions appear to have more potential to alter symptoms in CLBP than physically inactive interventions. Yet, the following four treatment modalities are not recommended due to lack of qualitative evidence and/or conflicting evidence: back schools, sensory discrimination training, proprioceptive exercises, and sling exercises [26,30,34,35]. Therefore, based on current evidence, these types of therapy should not be considered for CLBP management. 

The other active therapies for CLBP listed in Table 1 can be subdivided in physiotherapeutic treatment modalities that include a psychological component (i.e., multimodal), and treatment modalities that focus purely on physical exercises and movements. All included exercise modalities (aerobic exercise, strength/resistance exercise, coordination/stabilization exercise, motor control, and pilates) can effectively reduce pain and disability compared to minimal, passive/conservative, or no intervention [24,29,31,33]. However, when compared to each other (or to other active treatments), no differences can be found between different exercise modalities [24,29,31,33]. This is at odds with evidence in healthy people, where—for example—resistance training can reduce pain sensitivity to a greater extent than aerobic exercise [55]. 

Taken together, the information available regarding exercise interventions in CLBP and the wide variety in duration, intensity and methods of training, we cannot recommend which groups or types of exercise interventions are most effective [24,29,31,33]. From a motivational point of view, we recommend taking the patient’s preferences and abilities into account when deciding upon exercise modalities to use. Interestingly, when exercise therapy reduces pain and disability in people with CLBP, the improvements are often unrelated to an improvement in physical function [56]. Therefore, it is suggested that other exercise-induced changes like improved psychological status and cognitions (e.g., reduced anxiety, catastrophizing, and fear) influence pain and disability more than changes in physical function. This might explain the difficulties currently encountered to identify the optimal exercise modality and dosage for CLBP management [24,57]. This statement is (partly) underscored by the evidence on treatment modalities that combine exercises with a psychological component (i.e., biopsychosocial approach) [20,23,32].

Three systematic reviews (two of which included a meta-analysis) focused on the effectiveness of a biopsychosocial treatment approach [20,23,32]. This approach involves a physical component combined with a psychological component and/or a social/work targeted component [32]. Results of this approach compared to other active treatments are promising. For example, while there was no difference at short- and intermediate-term follow-up, behavioral psychological interventions were more effective to reduce pain at short-term and long-term follow-up than active treatments without a psychological component [20,58,59]. Interestingly, the best results were found for multidisciplinary biopsychosocial rehabilitation [32]. Importantly, the systematic review and meta-analysis investigating this rehabilitation approach does not allow to differentiate whether the positive results emanate from the multidisciplinary approach, the biopsychosocial focus, or both, as comparator studies all involved a monodisciplinary biomedical approach (e.g., electrotherapy, aerobic, stretching and strengthening exercises, traction, TENS, manual therapy, back school, surgery, etc.) [32]. Yet, both compared to monodisciplinary usual care and to monodisciplinary physical treatment, multidisciplinary biopsychosocial rehabilitation was found to be more effective to reduce pain and disability, even at long-term follow-ups [60,61,62,63,64,65,66,67]. These results indicate that a multidisciplinary biopsychosocial approach can provide CLBP patients with relevant tools to maintain positive treatment effects long term. This is underscored by evidence that the effect on work equates to a person having roughly double the odds of being at work after 12 months if they received a multidisciplinary rehabilitation program rather than a physical treatment alone [32,68,69,70,71]. Interestingly, studies focusing on the costs-effectiveness of interdisciplinary rehabilitation programs for chronic (pediatric) pain in general found significant reductions in medical costs post-treatment compared to the pretreatment phase [72,73,74].

Yet, a multidisciplinary approach can be time-consuming, and resource intensive. As there is currently no evidence available that directly compares a biopsychosocial approach in a monodisciplinary versus a multidisciplinary setting, future researchers should focus on the question if it is the multidisciplinary or rather biopsychosocial focus that explains these positive results. Interestingly, a large randomized controlled trial recently conducted by our group has investigated the effectiveness of a biopsychosocial approach (i.e., combining pain neuroscience education and cognition-targeted exercise therapy) delivered monodisciplinary by a physiotherapist only [75]. This approach was able to reduce pain, symptoms of central sensitization, and to improve psychophysiological measures of central sensitization, disability, pain cognitions, mental health and physical functioning (medium to large effect sizes) compared to an active control treatment. Using this example, we want to underscore that even in a monodisciplinary setting a biopsychosocial approach can be effective and should be targeted by clinicians. A treatment manual of this approach is published and can be accessed freely online (https://bit.ly/2WcA1re) [76].

The added value of a combined, biopsychosocial approach (i.e., adding psychological components to active physiotherapy treatments) is further underscored by a systematic review and meta-analysis that focused on the effectiveness of stand-alone psychological interventions for CLBP [37]. This review concluded that, compared to a waitlist, psychological interventions were superior to reduce pain intensity and improve quality of life, but showed equal results when compared to an active (i.e., exercise) control intervention [37]. 

Additionally, we would like to highlight the possible advantage of incorporating graded exposure techniques into the management of chronic low back pain. Graded exposure is a treatment modality that identifies feared exercises or activities, and exposes the patient to these exercises or activities in a hierarchical fashion, starting with an exercise or activity that elicits minimal amounts of fear and progressing only when this fear reduces [28]. One systematic review and meta-analysis focusses both on graded activity and graded exposure in nonspecific CLBP [28]. While graded activity can only improve disability when compared to a waitlist or usual care control group and does not show superior to other forms of exercises, there is some indicative research showing that graded exposure is more effective than graded activity to improve disability and catastrophizing short term [28]. However, currently there are no systematic reviews or meta-analyses available to allow firm conclusions on the potential of graded exposure in chronic low back pain management. Therefore, we suggest that clinicians can screen for the possible presence of feared movements and activities, and to tackle them using graded exposure techniques upon occurrence [77,78].

Last, we would like to underline a recent meta-analysis on the effectiveness of walking interventions [21]. When compared to education or other active exercises, walking improves pain, disability, quality of life and fear-avoidance to a similar extent. Therefore, walking interventions are not recommended as sole use, but given the low-budget and easy, accessible characteristics of walking, it can be a valuable home-based addition to other therapy modalities as it can increase physical activity, overcome activity avoidance, and minimize barriers for other types of exercise [21,22]. Walking at a low to moderate intensity imposes low risk of (musculoskeletal) injury and can improve aerobic capacity, body mass index, systolic/diastolic blood pressure, triglyceride levels, and high-density lipoprotein cholesterol levels in both healthy and sedentary individuals [22,79,80]. Therefore, clinicians should consider implementing walking exercises for CLBP management, when combined with other types of recommended, active treatment. 

### 2.4. International Guidelines

A critical review of LBP guidelines (2017) [81] used the Appraisal of Guidelines Research and Evaluation (AGREE) instrument to assess their quality and recommends four (out of 17 available) guidelines for LBP management [40,82,83,84]. Two of these guidelines (NICE guidelines and Dutch physiotherapy guidelines) focused on CLBP as a specific group apart from (sub)acute LBP [40,82] and will be discussed here. For the NICE guidelines, we refer to the updated version that was published in 2016. Additionally, the recommendations of two more recently published guidelines that were not yet included in the critical review will be discussed [85,86]. An overview of the recommendations included in these (clinical) guidelines can be found in Table 2. We will not discuss all recommendations in detail here but will rather highlight some striking features and parallels between guidelines. 

Although several differences exist between the different guidelines, exercise is recommended in all of them [40,82,85,86]. Interestingly, all of them also recognize that none of the exercise modalities is superior to the others: health care providers can choose any type of exercise (general, aerobic, strengthening, yoga, group-based or individual, etc.), but should specifically consider the patient’s preferences, needs and capabilities while choosing the exercise modality. The NICE guidelines even take it one step further and identify exercise as key treatment modality for LBP, given the recommendation to only consider manual therapy and/or psychological therapies if it is a part of a treatment package including exercise [40]. For multidisciplinary biopsychosocial rehabilitation—the intervention that shows high potential based on available systematic reviews and meta-analysis (see Table 1)—the NICE guidelines recommend considering this approach when significant psychosocial obstacles limit recovery, or when previous treatments have not been effective.

Importantly, these guidelines all agree not to recommend transcutaneous electrical nerve stimulation, interferential therapy (electrotherapy), or ultrasound for the treatment of CLBP. Other not-to-use modalities in CLBP management as identified in at least one of these guidelines are: traction, biofeedback, massage, laser therapy, taping, lumbar support, postural exercises, orthotics, and percutaneous electrical nerve stimulation. Interestingly, all modalities that are not recommended comprise physically inactive techniques, i.e., this implies lack of participation from the individual receiving the therapy intervention. This is in line with the conclusions made based on the systematic reviews (and meta-analysis) included in Table 1. The American Physical Therapy Association (APTA) even warns us of the negative effects of applying physically inactive treatments for any type of patient: these treatments can delay recovery and lead to poor long-term outcomes by reinforcing a passive role, promoting inactivity and disability behavior, and ‘medicalizing’ the patient [87]. Given the ‘active’ focus of recommended treatment modalities, this advice should also be taken into consideration when treating patients with CLBP. While physically inactive treatments (like manual therapy) appear to have potentially positive effects, they should not be used as sole treatment but rather in a multimodal approach focusing mainly on activating the patient [40]. 

## 3. Promising Directions for Clinical Practice

**Figure 1:**
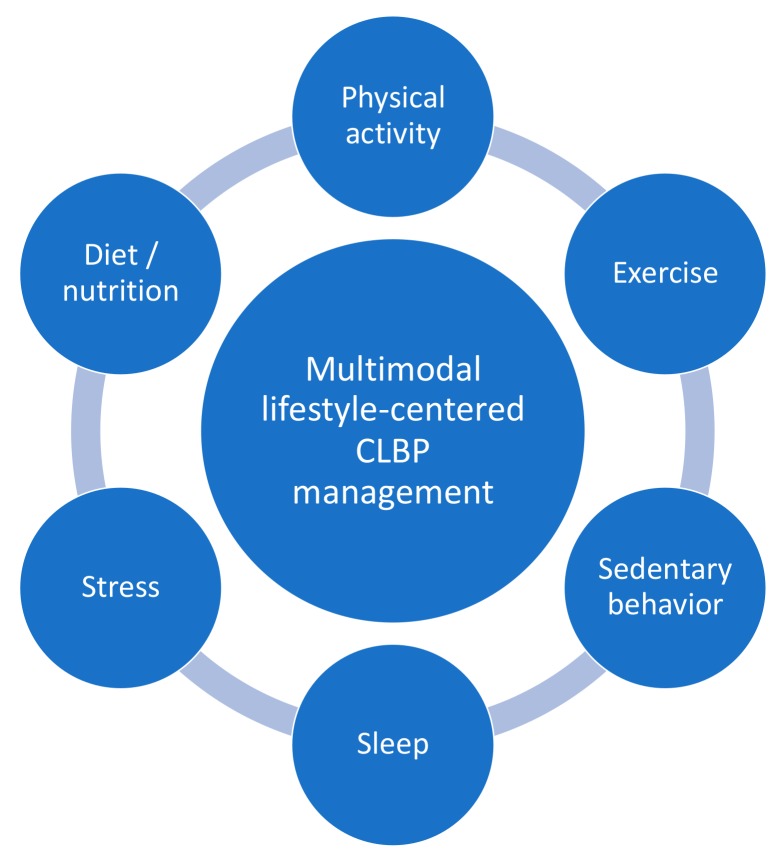
Promising direction for further research: a multimodal lifestyle-centered approach for people with chronic low back pain (CLBP).

Over the past decades, scientific understanding of CLBP has increased substantially. This has shifted treatment approaches away from pure biomedical treatments to multimodal approaches that acknowledge the complex biopsychosocial nature of CLBP. The latter includes addressing lifestyle factors, like physical activity and sedentary behavior, exercise, stress, sleep, and nutritional aspects (Figure 1). In the general chronic pain population, the influence of lifestyle factors like (chronic) stress, insomnia and sleep problems, depression, smoking, alcohol, obesity and nutrition are already acknowledged [88,89,90,91,92]. Additionally, the overview of best evidence non-invasive rehabilitation for CLBP in this paper highlights the importance of physical activity and exercise therapy for CLBP management. Still, within CLBP management specifically, other lifestyle factors have received little attention in scientific literature so far. Yet, a multimodal lifestyle-centered approach (Figure 1) could lead to a long-term decrease of the psychological and socio-economic burden of chronic pain. 

Incorporating stress management in CLBP treatment could help patients to cope with everyday stressors, and leads to a clinically meaningful reduction in disability even at long-term (one year) follow-up [93]. Stress management can help to increase acceptance of physical discomfort and difficult emotions [93]. The advantage of a multimodal approach that addresses different lifestyle factors can be underscored by the interconnection between stress and sleep in people with CLBP [94]. Numerous studies report a strong association between anxiety levels and insomnia severity [95,96], and daily life stress can negatively impact sleep [97]. As poor sleep acts as a precipitating and perpetuating factor [98], and can represent a barrier for effective chronic pain management [99], its importance for CLBP management is evident. Additionally, people with chronic pain will spontaneously engage in more physical activity following a better night of sleep [100], again underscoring the importance of a multimodal lifestyle-centered approach.

Similar to sleep, overweightness and obesity are risk factors for developing LBP, and are associated with more severe and debilitating pain as pain intensity and disability show dose-responses to Body Mass Index, waist circumference, percent fat, and fat mass [101,102,103,104,105,106,107]. Unfortunately, overweight and obesity are an often overlooked lifestyle factor of importance in CLBP, while overweight/obese people with CLBP are likely to have more complex needs requiring a focus on lifestyle factors [108]. A recent study examining a nonsurgical weight loss program (i.e., physical exercise plus changes in dietary behavior) found that people with CLBP (*n* = 46) not only lost body weight, but also experienced less pain and disability [109]. Given the uncontrolled nature of this study, methodologically-sound randomized controlled trials examining the added value of such an approach are needed. Yet, if therapists were to implement a weight reduction program for CLBP management, this program should include changes in diet, behavior and physical activity [109], given the American College of Sports Medicine Position Stand that a moderate dietary restriction combined with a physical activity program (i.e., a deficit of 500 to 700 kcal on the energy balance) is effective and delivers long-term results [110].

Importantly, such multimodal lifestyle approach should primarily be implemented as a patient-centered approach, tailoring the included treatment aspects to the preferences and attitudes of the individual. This includes continuous (non-)verbal communication, education during all aspects of treatments, patient-defined goals, patient-empowerment, and a confident therapist who has sufficient social and interpersonal skills and shows specific knowledge [111]. To optimize the success rate of this approach, principles of self-monitoring, goal setting and feedback can also be integrated [112,113]. Given the need for behavioral changes in such a lifestyle approach, motivational interviewing techniques can help the therapist to overcome difficulties experienced by the patient to engage in this positive health behavior [114]. For example, consequences of an unhealthy lifestyle, as well as barriers for change, can be discussed, together with examples of how a better lifestyle can impact pain and quality of life, including a plan-of-action [115]. Motivational interviewing aims to develop autonomous motivation in the patient by increasing perceived competence, self-regulation and self-efficacy [115]. As higher self-efficacy is one of the key factors associated with better treatment outcome in chronic pain, motivational interviewing techniques are useful to consider even beyond CLBP management [116,117]. Clinicians and researchers should focus on this multimodal approach to CLBP to aim for long-term improvements in pain, disability and quality of life, rather than a short-term relief. As this approach could increase the empowerment of the patient and thus increase their personal control over the symptoms, the need for constant follow-up and supervision of a physiotherapist—and the related socio-economic costs—could be diminished. 

## 4. Conclusions

Given the high prevalence of CLBP, and the overwhelming evidence available on its possible management, this paper aimed to give a clear overview of best evidence practice. To conclude, most physically inactive therapies should not be considered for CLBP management, except for pain neuroscience education and spinal manipulative therapy if combined with exercise therapy, with or without psychological therapy. Regarding active therapy, back schools, sensory discrimination training, proprioceptive exercises, and sling exercises should not be considered for CLBP management due to a lack of qualitative evidence and/or conflicting evidence. Exercise interventions, on the other hand, are recommended, but while all exercise modalities appear effective compared to minimal, passive/conservative or no intervention, there is no evidence that some specific types of exercises are superior to others. Therefore, we recommend choosing exercise modalities according with the patient’s preferences and abilities. When combining exercise interventions with a psychological component, effects are better than an approach without psychological component and remain at long term. 

**Key messages for CLBP rehabilitation**

- Do not consider the use of therapeutic ultrasound, kinesiotape, transcutaneous electrical nerve stimulation, massage and osteopathic interventions.
- Pain neuroscience education and spinal manipulative therapy can have positive effects but should not be used as stand-alone treatment. Consider these modalities only as part of a treatment package including exercise, with or without psychological therapy.
- Do not consider back school, sensory discrimination training, proprioceptive exercises, and sling exercises.
- Exercise therapy is highly recommended, but it is not clear which duration, intensity and methods of training are best.
- Consider a combined physical and psychological intervention incorporating cognitive behavioral techniques to maintain positive effects at long-term.

## Figures and Tables

**Table 1:** Best evidence table for non-invasive rehabilitation in people with chronic low back pain: evidence from systematic reviews and meta-analyses.

Author, Year	LoE	Intervention and Sample	Main Outcomes and Results	Mono-/Multi-/Transdisciplinary [Involved Rehabilitation Professions]	Remarks	Recommended for Clinical Practice?
***Physically inactive interventions***						
Noori, 2019 [11]	1A	Therapeutic ultrasound (*n* = 333)	3 studies: ↓ pain after ultrasound compared to placebo or exercise 3 studies: no effect	Not stated [Not stated]	Small samples, most studies lack follow-up period. No meta-analyses.	Lack of strong evidence for the use of ultrasound (LoC 1)
Li, 2019 [12]	1A	Kinesiotape (*n* = 627) *Meta-analysis*	Pain intensity: No significant effect Disability: Significant ↓ in Oswestry Disability Index, but not in Roland Morris Disability Questionnaire	Monodisciplinary [Physiotherapist]	/	Lack of evidence for the use of kinesiotape (LoC 1)
Wood, 2019 [13]	1A	Pain Neuroscience Education (PNE) (*n* = 615) *Meta-analysis*	**PNE alone**: no significant change in pain, but significant ↓ in disability and kinesiophobia at short term compared to an alternative intervention. **PNE combined with other PT interventions**: significant ↓ in pain at short-term.	Monodisciplinary [Physiotherapist or general practitioners]	Heterogeneity in outcome measures.	Moderate quality evidence to use pain neuroscience education as adjunct to usual physiotherapy (LoC 1)
Resende, 2018 [14]	1A	Transcutaneous electrical nerve stimulation (TENS) (*n* = 575) *Meta-analysis*	Pain: Significant reduction during therapy, but not immediately after therapy or at 1 or 3mo follow-up. Disability: No effect during, or after therapy.	Not stated [Not stated]	Similar conclusion in other meta-analysis on effects of TENS on chronic back pain (Wu, 2018) [15]	Not recommended to use for CLBP (LoC 1)
Furlan, 2015 [16]	1A	Massage (*n* = 2548) *Meta-analysis*	**Compared to inactive control:** Massage may be more effective for pain and disability at short term. Conclusions at long term are unclear. **Compared to active control:** Results are unclear, no conclusions can be made at short-and long-term follow-up.	Not stated [Not stated]	Subacute and CLBP results are presented as one group. Very low quality of evidence.	Massage is not recommended to treat CLBP (LoC 1)
Orrock, 2013 [17]	1A	Osteopathic intervention (*n* = 330)	Similar effect of osteopathic intervention when compared to sham intervention or exercise and PT.	Monodisciplinary [Osteopath]	Only two studies available. No meta-analysis.	Not recommended due to lack of evidence (LoC 1)
Rubinstein, 2019 [18]	1A	Spinal manipulative therapy (*n* = 9211) *Meta-analysis*	Pain: Moderate evidence that spinal manipulative therapy provides statistically better results than other interventions (exercise, PT, back school, medical care) at 6mo, but not at 1 and 12mo follow-up. Function: Moderate quality evidence that spinal manipulative therapy provides a small, statistically better result than other interventions at 1mo, but not at 6 or 12mo follow-up.	Monodisciplinary [Physiotherapist, chiropractor, manual therapist, osteopath]	Many studies with high risk of bias.	Possible adjunctive therapy. Produces similar effects to recommended therapies. Possibility of adverse events. (LoC 1)
***Physically active interventions***						
Hajihasani, 2019 [19]	1A	Adding Cognitive Behavioral Therapy (CBT) to PT (*n* = 965)	**Compared to PT alone**: Pain: mixed results, significant ↓ in 5 out of 10 studies; Disability: mixed results, significant ↓ in 4 out of 7 studies; Quality of Life: mixed results, significant ↓ in 2 out of 5 studies; Depression: mixed results, 2 studies show no changes, while one study shows exacerbation of depressive symptoms after adding CBT.	Mono- or multidisciplinary [Psychologist and physiotherapist]	No meta-analysis.	Mixed results, no clear indication for adding CBT to PT (LoC 1)
Zhang, 2019 [20]	1A	Group-based physiotherapist-led behavioral psychological interventions (*n* = 1927) *Meta-analysis*	**Compared to waitlist or usual care**: Significant pain reduction at short-, intermediate, and long-term follow-up. **Compared to active treatment**: No difference between groups at short- or intermediate, but significant lower pain after behavioral therapy at long-term follow-up.	Monodisciplinary [Physiotherapist]	Heterogeneity in methods.	Yes, while there is no difference with active treatments at short and intermediate follow-up, behavioral treatments appear more effective at long-term follow-up. There are indications that the addition of behavioral components can reduce sick leave.
Vanti, 2019 [21]	1A	Walking interventions (*n* = 510) *Meta-analysis*	Pain, disability, quality of life and fear-avoidance improve equally by walking or exercise.	Not stated [Physiotherapist]	Same conclusion in similar meta-analysis by Sitthiporn-vorakul, 2018 [22]	Walking is not more effective for reducing pain and disability compared to exercise or education, but can be used as a low-budget and easy accessible alternative (LoC 1)
Van Erp, 2018 [23]	1A	Primary Care Interventions Using a Biopsychosocial Approach (*n* = 1426)	**Compared to education/advice**: Functional disability ↓ at short, mid and long term; Pain ↓ at short, mid and long term; Quality of life: No differences **Compared to physical activity therapy**: Functional disability: No differences; Pain: mixed results, 2 out of 4 studies report significant ↓ in pain in biopsychosocial approach	Mono- or multidisciplinary [Physiotherapist, combined with nurses, psychologist, or occupational therapist]	Heterogeneity in study and treatment designs. No meta-analysis.	Use of bio-psychosocial interventions in primary care is beneficial over education and advice (LoC 1)
Wewege, 2018 [24]	1A	Aerobic and resistance exercise interventions (*n* = 322) *Meta-analysis*	**Pooled results of aerobic and resistance training**: Small significant improvement in pain and a trend towards significance for decreased disability and improved mental health. No differences were found for physical health (SF36).	Monodisciplinary [Physiotherapist or exercise therapist]	/	Moderate quality evidence for the use of aerobic and resistance training (LoC 1)
Luomajoki, 2018 [25]	1A	Movement control exercise therapy (*n* = 781) *Meta-analysis*	**In global group**: Short-term ↓ in disability, but not in pain compared to active control treatment. No long-term effects. **In subgroup with movement control impairment**: Short- and long-term ↓ in pain and disability.	Monodisciplinary [Physiotherapist]	Small sample sized and heterogeneity of included studies.	Very low to moderate quality of evidence to use movement control exercises in CLBP AND movement control impairment (LoC 1)
Parreira, 2017 [26]	1A	Back School (*n* = 4105) *Meta-analysis*	Pain: Low quality of evidence for reduction at short term, but not at intermediate or long-term follow-up compared to no treatment. Disability: Low quality of evidence that back schools are not effective at intermediate or long-term follow-up compared to no treatment.	Monodisciplinary [Physiotherapist or medical specialist]	Low quality of evidence	Because of low quality of evidence, back schools are not recommended for CLBP (LoC 1)
Du, 2017 [27]	1A	Self-management (*n* = 2188) *Meta-analysis*	Pain: Significant reduction using self-management at immediate, short-term, intermediate and long-term follow-up compared to a control intervention. Disability: Significant reduction using self-management at immediate, short-term, intermediate and long-term follow-up compared to a control intervention.	Mono- or multidisciplinary, and/or internet-based [Physiotherapist, psychologist, exercise therapist, and/or internet-based]	/	Yes, there is moderate-quality evidence that self-management has a moderate effect on pain intensity, and small to moderate effect on disability (LoC 1)
López-de-Uralde-Villanueva, 2016 [28]	1A	Graded Activity and Graded Exposure (*n* = 1486) *Meta-analysis*	**Graded activity vs other forms of exercises**: No difference for disability, quality of life or pain at any time-point. **Graded activity vs waitlist or usual care**: Graded activity is more effective to reduce disability, but not pain at short- and long-term follow-up. **Graded activity vs graded exposure**: Graded exposure was more effective to reduce disability and catastrophizing in the short term. There is no difference between both regarding the effect on pain.	Not stated [Not stated]	Poor methodological quality of many included studies. Possible publication bias could not be assessed.	There is limited evidence that graded activity significantly reduces disability in the short and long term compared to a control intervention, but not when compared to an active control intervention. There is strong evidence that graded activity cannot change pain in the short, intermediate, and long term compared to a control intervention. There are indicative findings that graded exposure is better than graded activity at decreasing disability and catastrophizing in the short term. (LoC 1)
Saragiotto, 2016 [29]	1A	Motor control exercise (*n* = 2431) *Meta-analysis*	**Compared to other exercises**: Small, but not clinically important effect on pain and disability at short term, but not at intermediate or long-term follow-up. **Compared to manual therapy**: No effect on pain and disability. **Compared to minimal intervention**: Clinical important effect on pain at short- and long-term. Small, but not clinically important effect on disability at short- intermediate and long-term.	Not stated [Not stated]	/	Motor control exercises are more effective than a minimal intervention, but is not more effective than other forms of exercise or manual therapy (LoC 1)
Kälin, 2016 [30]	1A	Sensory discrimination training (*n* = 255)	Both sensory discrimination and control treatments (TENS, back school, sham treatment) led to a decrease in pain and an improvement in function.	Monodisciplinary [Physiotherapist]	Conflicting evidence, low quality of included studies. No meta-analysis.	Conflicting evidence, no clear conclusion or recommendation possible (LoC 1)
Yamato, 2015 [31]	1A	Pilates (*n* = 510) *Meta-analysis*	Pain: Pilates is more effective at short and intermediate term compared to minimal intervention, but not compared to other exercise interventions. Disability: Pilates is more effective at short and intermediate term compared to minimal intervention, but not compared to other exercise interventions.	Monodisciplinary [Pilates instructor]	Although the review focused on (sub)acute and chronic LBP, but all included studies dealt about CLBP.	Pilates is more effective than minimal intervention (low- to moderate quality of evidence), but there is no evidence for the superiority of Pilates to other forms of exercise (LoC 1)
Kamper, 2015 [32]	1A	Multidisciplinary biopsychosocial rehabilitation (*n* = 6858) *Meta-analysis*	**Compared to usual care**: Multidisciplinary biopsychosocial rehabilitation is more effective to reduce pain and disability, even at long-term. **Compared to physical treatment**: Multidisciplinary biopsychosocial rehabilitation is more effective to reduce pain and disability, even at long-term.	Multidisciplinary [Physical, psychological, educational, and/or work-related components delivered by expert healthcare providers]	Clinical heterogeneity among included studies.	Yes, multidisciplinary biopsychosocial rehabilitation is more effective than usual care or physical treatment (LoC 1)
Searle, 2015 [33]	1A	Exercise interventions (*n* = 4462) *Meta-analysis*	**General comparison**: Exercise has a small but significant benefit for the treatment of non-specific CLBP and is more effective than conservative therapies (wait list or usual activities, general practitioner care, electrotherapies and manipulative therapies). **Sub-analysis**: Strength/resistance, coordination/stabilization, and combined exercise is more effective than conservative therapies, but not cardiorespiratory exercise.	Not stated [Not stated]	Heterogeneity in application of exercise interventions.	Yes. Beneficial effect of strength/resistance and coordination/stabilization exercise programs over other interventions (LoC 1)
McCaskey, 2014 [34]	1A	Proprioceptive exercises (*n* = 1380)	**Perceptual proprioceptive training**: More effective for pain reduction than back school. Two studies, very low quality of evidence. **Joint repositioning training**: More effective for short-term pain reduction than no intervention. No difference with other exercises. Low quality of evidence. **Multimodal proprioceptive training**: More effective for short-term pain reduction than no intervention. No difference with other exercises. Low quality of evidence.	Monodisciplinary [Physiotherapist]	Overall low quality of evidence. No meta-analysis.	No consistent benefit in adding proprioceptive exercises for CLPB rehabilitation (LoC 1)
Yue, 2014 [35]	1A	Sling exercise (*n* = 706) *Meta-analysis*	Sling exercises are not more effective for improving pain or function compared to other forms of exercise.	Not stated [Not stated]	Low quality of included studies.	Based on the available evidence, sling exercises are not recommended (LoC 1)
Holtzman, 2013 [36]	1A	Yoga (*n* = 851) *Meta-analysis*	Pain and disability improved directly post-treatment (moderate to large effect sizes) and remained at long-term follow-up (small to medium effect sizes). Effects were compared to no treatment/waitlist, stretching, usual care, education and exercise.	Monodisciplinary [Yoga therapist]	Heterogeneity in yoga interventions. High quality of included studies.	Yes, possible adjunctive to PT intervention (LoC 1)
Hoffman, 2007 [37]	1A	Psychological interventions (*n* = 1747) *Meta-analysis*	**Compared to waitlist**: Psychological interventions are superior to reduce pain intensity and health-related quality of life. **Compared to active control (e.g., treatment as usual) intervention**: Psychological interventions are not superior	Mono- or multidisciplinary [Not stated]	/	Psychological interventions are more effective than no intervention, but not compared to active interventions (LoC 1)

Level of Evidence (LoE): 1A: Systematic review of randomized controlled trials; 1B: Individual randomized controlled trials; 2A: Systematic review of cohort studies; 2B: Individual cohort study or low quality randomized controlled trials; 3A: Systematic review of case-control studies; 3B: individual case-control study; 4: Case-series; 5: Expert opinion. Level of Conclusion (LoC): LoC 1: Research of evidence level 1A or at least 2 independent conducted studies of evidence level 1B; LoC 2: 1 research of evidence level 1B or at least 2 independent conducted studies of evidence level 2B or 3B; LoC 3 1 research of evidence level 2B, 3B or 4; LoC 4: Opinion of experts or Inconclusive or inconsistent results between various studies. Abbreviations: LoE = Level of Evidence; LoC = Level of Conclusion; PT= physiotherapy; CLBP = Chronic Low Back Pain.

**Table 2:** Overview of recommendations in (clinical) guidelines for chronic low back pain management.

Guideline	Recommendation for CLBP	
**Bekkering et al**. Dutch Physiotherapy Guidelines for Low Back Pain (2003) [82]	Recommended	- **Exercise therapy (not clear which exercises are best):** Strong evidence that exercise therapy is equally effective compared to passive physiotherapy techniques. Strong evidence that exercise therapy is more effective than standard care by the general practitioner. - **Behavioral treatment: may be useful.** Strong evidence for a moderately positive effect on pain compared to no treatment, waitlist or placebo. Effectiveness compared to other treatments not clear.
	Not recommended	- **Traction** - **Biofeedback** - **Massage** - **Transcutaneous electrical nerve stimulation** - **Ultrasound** - **Electrotherapy** - **Laser therapy**
**Wong et al**. Clinical guidelines for the noninvasive management of low back pain (2016) [86]	Recommended	- **Education**: Advice and information promoting self-management, evidence-based information on expected course and effective self-care options, advice to stay active. - **Exercises**: No recommendations for or against any specific type of exercise, consider patient preferences. - **Manual therapy, including spinal manipulation** - **Multimodal rehabilitation**: including physical and psychological interventions (e.g., cognitive/behavioral approached and exercise). - **Recommended by some guidelines**: Massage, acupuncture, antidepressants.
	Not recommended	- **Muscle relaxants** - **Gabapentin** - **Passive modalities**, including transcutaneous electrical nerve stimulation, laser, interferential therapy and ultrasound.
**Qaseem et al**. Noninvasive treatments for acute, subacute and chronic low back pain (2017) [85]	Recommended	- **Exercise**: Moderate-quality evidence for small improvements in pain relief and function when compared to no exercise or usual care. No evidence on which exercise regimen is best. - **Motor control exercise**: Low-quality evidence for the effectiveness of motor control exercise (small improvements in pain and function) compared to minimal intervention, general exercise, and multimodal physical therapy. Low quality of evidence found no differences between motor control exercises plus exercise or exercise alone. - **Tai Chi**: Low-quality evidence showed that Tai Chi results in moderate pain reduction compared to waitlist or no intervention. - **Yoga**: Low-quality evidence showed that yoga results in a small pain reduction compared to exercise. - **Psychological therapies**: Low-quality evidence showed positive effects of progressive relaxation therapy, mindfulness relaxation, electromyography biofeedback training, operant therapy and cognitive behavioral therapy compared to waitlist. Low-quality evidence shows no difference between psychological therapies and exercise or physical therapy, and no difference between psychological therapies plus exercise and exercise alone. - **Multidisciplinary rehabilitation**: Moderate-quality evidence for effectiveness to improve pain and disability compared to usual care, no treatment, or physical therapy.
	Not Clear	- **Pilates** - **Acupuncture**: Low-to-moderate-quality evidence shows effectiveness of acupuncture but compared to no treatment or sham treatment. No improvement found in function. - **Spinal manipulation** - **Low-level laser therapy**
	Not recommended	- **TENS** - **Ultrasound** - **Lumbar support** - **Taping**
**National Guideline Centre**. NICE Guideline Low back pain and sciatica (2016) [40]	Recommended	- **Self-management**: Provide advice and information tailored to the patient’s needs and capacities, including information on the nature of the pain, and encouragement to continue normal activities. - **Exercise**: Consider group exercise programs, take into account the patient’s specific needs, preferences and capabilities when choosing the type of exercise. - **Manual therapy (spinal manipulation, mobilization or soft tissue techniques)**: Can be used, but only as part of a treatment package including exercise, with or without psychological therapy. - **Cognitive behavioral therapy**: As part of a treatment package, including exercise, with or without manual therapy. - **Multidisciplinary biopsychosocial rehabilitation**: Consider a combined physical and psychological intervention incorporating cognitive behavioral techniques when significant psychosocial obstacles limit recovery, or when previous treatments have not been effective. - **Return to work**: Promote and facilitate. - **Normal activities of daily living**: Promote and facilitate.
	Not recommended	- **Opioids** - **Postural exercise or education** - **Orthotics, belt, corsets or rocker sole shoes** - **Traction** - **Acupuncture** - **Ultrasound** - **Percutaneous electrical nerve stimulation** - **Transcutaneous electrical nerve stimulation** - **Interferential therapy**
